# Principles of proteome allocation are revealed using proteomic data and genome-scale models

**DOI:** 10.1038/srep36734

**Published:** 2016-11-18

**Authors:** Laurence Yang, James T. Yurkovich, Colton J. Lloyd, Ali Ebrahim, Michael A. Saunders, Bernhard O. Palsson

**Affiliations:** 1 Department of Bioengineering, University of California, San Diego, La Jolla, California, USA; 2 Bioinformatics and Systems Biology Program, University of California, San Diego, La Jolla, California, USA; 3 Department of Management Science and Engineering, Stanford University, Stanford, California, USA; 4 Novo Nordisk Foundation Center for Biosustainability, The Technical University of Denmark, Hørsholm, Denmark

## Abstract

Integrating omics data to refine or make context-specific models is an active field of constraint-based modeling. Proteomics now cover over 95% of the *Escherichia coli* proteome by mass. Genome-scale models of Metabolism and macromolecular Expression (ME) compute proteome allocation linked to metabolism and fitness. Using proteomics data, we formulated allocation constraints for key proteome sectors in the ME model. The resulting calibrated model effectively computed the “generalist” (wild-type) *E. coli* proteome and phenotype across diverse growth environments. Across 15 growth conditions, prediction errors for growth rate and metabolic fluxes were 69% and 14% lower, respectively. The sector-constrained ME model thus represents a generalist ME model reflecting both growth rate maximization and “hedging” against uncertain environments and stresses, as indicated by significant enrichment of these sectors for the general stress response sigma factor σ^S^. Finally, the sector constraints represent a general formalism for integrating omics data from any experimental condition into constraint-based ME models. The constraints can be fine-grained (individual proteins) or coarse-grained (functionally-related protein groups) as demonstrated here. This flexible formalism provides an accessible approach for narrowing the gap between the complexity captured by omics data and governing principles of proteome allocation described by systems-level models.

Genome-scale models have been used to conduct systems-level studies of cellular metabolism for over 15 years^1^. They can elucidate structures in large datasets that are not captured by purely statistical models^2^. In particular, the COnstraint-Based Reconstruction and Analysis (COBRA) field has a rich history of using omics data to refine and improve predictions^3^,^4^,^5^. These methods have been useful for the systems biology community. For example, tissue-specific models could be generated for health applications^6^ or computational strain design could be improved for metabolic engineering^7^. Despite many methods for omics integration in COBRA, the general problem of relating gene expression to metabolic flux and cell physiology remains challenging. One challenge has been that metabolic models only indirectly relate expression to flux. ME (Metabolism and macromolecular Expression) models^8^,^9^,^10^,^11^ now relate gene and protein expression directly to metabolic flux. Therefore, in theory it is possible to integrate transcriptomics and proteomics data directly into COBRA to refine predictions. However, the ME model is multiscale and spans multiple cellular processes including metabolism and protein expression machinery. The latest ME model^11^ models the function of 1,678 genes described by nearly 80,000 reactions and 70,000 constraints involving biochemical species and macromolecules spanning nearly 70 cellular subsystems. Therefore, it is not obvious how changes to one part of the system affect others. Specifically, it is not obvious how expression changes for multiple proteins will quantitatively affect growth rate or metabolic fluxes. As a first step, a recent study shows that growth rate predictions are indeed markedly improved when the overall fraction of unused protein (i.e., expressed but not actively used) is constrained using estimates from proteomics data^12^. A remaining question then is whether constraining specific functional protein groups can also improve growth and metabolic flux predictions.

Schmidt *et al*. recently published a proteomics data Resource^13^ covering ~55% of the predicted *E. coli* genes (>95% by mass) under 22 experimental conditions. Using genome-scale models, we show how such proteomics resources can be used to reveal principles underlying proteome allocation. ME models compute growth optimal proteomes consistent with laboratory evolved strains accurately^14^, but are unable to compute processes that are not directly related to growth (e.g., stress response, preparation for unfavorable conditions)^15^. In anticipation of environmental change, generalist (wild-type) *E. coli* allocate a fraction of the proteome to non-growth related functions. Collectively, such allocation can be viewed as “hedging” against unknown enviromental challenges^14^, reflecting the evolutionary history of the organism and its successful survival strategy. Recent studies have estimated that 20% of the expressed proteome confers no direct fitness benefit^15^.

Elucidating trade-offs between multiple cellular objectives is an active field of systems biology research^16^,^17^. Here we develop a pragmatic approach for modeling the proteome allocation resulting from such complex cellular objectives following in the spirit of omics integration with genome-scale models. Namely, we define sector constraints using proteomics data. We then show that sector-constrained ME models can compute the proteome composition of generalist *E. coli* in a variety of different growth environments. We then compare the “optimal” versus the “generalist” proteomes to reveal principles underlying proteome allocation.

## Results

### Modeling generalist *E. coli* proteome allocation and physiology

We first computed optimal proteome allocation that maximized growth rate. Measured proteome allocation differed notably from these computed optimal proteomes. While the interactions between thousands of individual proteins is complex, the *E. coli* proteome has been shown to exhibit relatively simple relationships when proteins were grouped into meaningful “sectors” (e.g., linear relations with growth rate)^18^. Similarly, we coarse-grained the proteome into functional sectors. Here, we specifically used Clusters of Orthologous Groups^19^ (COGs) as they represent a reasonable trade-off between complexity (24 sectors) and protein function coverage.

**Figure 1:**
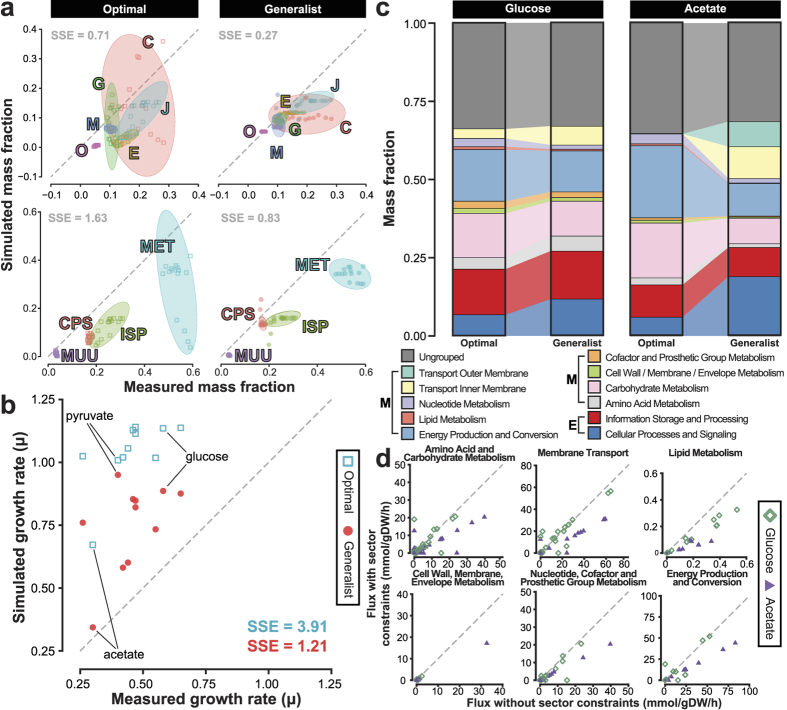
Model-based interpretation of proteomic data. (**a**) Predicted mass fractions are validated by proteins grouped by COG for optimal and generalist ME models. Ellipses show 95% confidence intervals. (**b**) Growth rate predictions improve due to proteome sector constraints. (**c**) The model predicts global proteome reallocation due to sector constraints for Metabolic (M) and Expression (E) systems. (**d**) The ME model computes global metabolic shifts due to proteome reallocation. Abbreviations: C, Energy production and conversion; E, Amino acid transport and metabolism; G, Carbohydrate transport and metabolism; J, Translation, ribosomal structure and biogenesis; M, Cell wall/membrane/envelope biogenesis; O, Posttranslational modification, protein turnover, chaperones; CPS, Cellular Processes and Signaling; ISP, Information Storage and Processing; MET, Metabolism; MUU, Mobilome, Unknown, and Ungrouped; SSE, sum of squared errors.

We then identified proteome sectors whose measured mass fractions were greater (over-allocated) and smaller (under-allocated) compared to the optimal proteomes across growth conditions (Fig. 1a). For our analysis we focused on the 15 minimal medium growth conditions under batch and chemostat culture without additional pH, osmotic or temperature stresses. Six COG sectors had high measured mass fractions (5% or more) under all 15 conditions. The minimum mass fraction across conditions for these COGs ranged from 5.4% to 16%, totaling 58% (Table 1). Meanwhile, the optimal proteomes allocated between 13% to 54% of proteome to the sum of these sectors (Fig. 1a). Additionally, the computed growth rates corresponding to optimal proteomes were consistently higher than measured (Fig. 1b).

We hypothesized that by constraining the ME model to more accurately allocate proteome to these large sectors, we could better predict growth rate and metabolism of the wild-type, generalist *E. coli*. To this end, we added new “sector constraints” to the ME model (constraint (5) in Methods). Here we constrained the sum of protein mass fractions within each of the six sectors; however, the formulation is general in that any individual protein or different sector definition (coarser or finer-grained) can be used. It is important to note that while our sector constraints involved 966 of the 1678 genes in the iJL1678 ME model, only six actual constraints were added to the model–only the sum of mass fraction of each sector was constrained. Therefore, the individual protein mass fractions were still computed by the ME model. Because our objective was to develop a generalist ME model, we used these coarse-grained sector constraints to prevent over-fitting the proteome to specific conditions.

The addition of the six sector constraints led to markedly improved agreement in growth rate predictions across all conditions, with 69% lower sum of squared error (SSE), overall (Fig. 1b and Supplementary Table S2). We also compared measured and computed proteome allocation for a functional grouping of proteins that was different from the COGs used for sector constraints. The SSE was 49% lower for proteome allocation (Fig. 1a).

We thus designated this sector-constrained ME model the Generalist ME model. Because the total proteome is limited in size, the increased allocation to certain sectors would lead to decreased resource allocation to at least one other sector. Thus, the sector constraints reflect costs of cellular decisions to over-allocate proteomic resources for purposes other than maximal growth on a minimal medium. For example, the COG categories “carbohydrate transport and metabolism”, “energy production and conversion”, and “translation, ribosomal structure and biogenesis” were enriched for control by the stress response sigma factor *σ*^S^ (Supplementary Table S1) and could reflect preparation for unfavorable conditions^15^.

### Proteomic and metabolic consequences of proteome sector constraints

Next, we examined the resource allocation of each computed proteome by metabolic (M) and macromolecular expression (E) model subsystem (Fig. 1c and Supplementary Fig. S1). Note that these (M) and (E) subsystems differ from the gene categorization used to define proteome sector constraints (i.e., COGs) in Table 1. Allocation to membrane transport proteins (specifically amino acid and carbohydrate transport and metabolism) was increased according to the sector constraints, although they had no fitness benefit for growth on acetate according to the optimal model (Supplementary Fig. S2). These sectors included genes controlled by the stress response sigma factor *σ*^S^. These sector constraints thus resemble foraging and stress response that intensifies in lower-quality substrates such as acetate^20^. The generalist acetate proteome was lowered in “energy production and conversion”. This sector showed considerable decrease in enzymes catalyzing acetate consumption (acetate kinase, phosphotransacetylase) and energy metabolism (cytochrome oxidase bo3, ATP synthase; see Supplementary Fig. S3), leading to a decreased growth rate. The optimal proteomes represent the minimal proteomic resources needed to grow at sub-optimal growth rates (Supplementary Fig. S4). The extent to which *E. coli* allocates its proteome beyond the minimum required was surprisingly high: a growth rate of 0.12 h^−1^ could theoretically be supported with 95% less proteome allocated to all M and E sectors than measured (Supplementary Table S3 and Supplementary Fig. S5).

The sector constraints also altered metabolic flux distributions (Fig. 1d and Supplementary Fig. S6). Specifically, proteome constraints induced statistically significantly smaller (*P* < 0.05) fluxes in 5 of 8 of the subsystems for glucose (Lipid Metabolism; Cell Wall/Membrane/Envelope Metabolism; Nucleotide, Cofactor and Prosthetic Group Metabolism; Amino Acid and Carbohydrate Metabolism; Other), and all 8 metabolic subsystems for acetate (Supplementary Table S4).

The sector constraints also affected predicted protein fraction of cell dry weight. Compared to the Optimal model, the Generalist model predicted 5.5% to 24.8% higher protein fraction (Table 2), which was significant (Wilcoxon rank sum test, *P* = 6.4 × 10^−9^). Interestingly, the Generalist model showed a clear and significant negative correlation between total proteome size and growth rate, whereas the Optimal model did not (Supplementary Fig. S7). This linear trend in the Generalist model arises because many of the sector constraints force expression of unused protein^12^ and is consistent with a previously observed growth rate-dependent decrease in constitutively expressed proteins^21^.

In addition, the Generalist model’s proteome size of 72.7% dry weight at dilution rate of 0.12 h^−1^ agrees well with the measured value of 70.1% for this dilution rate^22^. However, in unlimited growth on glucose, the Generalist model overestimates proteome size (62.4% versus 51.1% measured^22^). This result is due to the sector constraints being defined based on multiple carbon sources, whereas *E. coli* is more adapted for growth on its preferred substrate, glucose^23^.

### Validation of intracellular fluxes

We next validated intracellular flux predictions for the Optimal and Generalist models using metabolic flux analysis (MFA) data by Gerosa *et al*.^24^ for 7 carbon sources: acetate, fructose, galactose, glucose, glycerol, pyruvate, and succinate. The Generalist model was more consistent with MFA for 5 of 7 conditions (Supplementary Fig. S8). In particular, acetate, succinate and glycerol predictions improved greatly, with 68%, 41%, and 15% lower RMSE (root mean squared error), respectively. RMSE was higher for the Generalist model for glucose and galactose conditions (8.0% and 30% higher RMSE, respectively). However, when we performed similar validation using a different MFA data set^25^, we observed slightly (6.5%) lower RMSE on glucose and slightly (6.4%) higher RMSE for galactose (Supplementary Fig. S9). This discrepancy between MFA data sets partially arises because the MFA data relied on simplified models of central carbon metabolism, whereas the ME model considers the genome-scale metabolic network. The sector constraints most affected TCA cycle fluxes, with the Generalist model fluxes decreasing between 100% to 16.8% across conditions (Supplementary Data S1). Therefore, respiratory capacity was predicted to be most strongly affected by allocating proteome toward hedging functions.

### Sensitivity of predictions to parameter uncertainty

**Figure 2:**
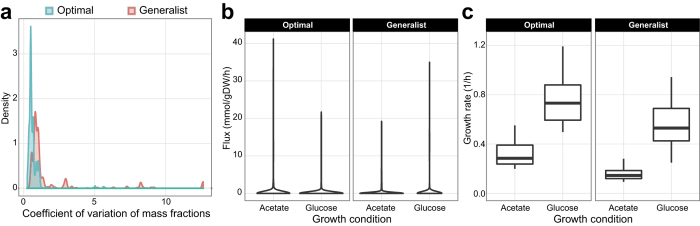
Sensitivity to model parameters. (**a**) Probability density of the coefficients of variation of simulated protein mass fractions across random perturbations of effective rate constants. (**b**) Variation in simulated metabolic fluxes upon perturbing effective rate constants. (**c**) Variation in simulated growth rate upon perturbing effective rate constants.

Due to proteome constraints, ME models exhibit essentially no flux variability at the proteome level at maximum growth rate^26^. However, optimal solutions can vary due to uncertainty in effective rate constants. Thus, we next assessed how sensitive growth rate and protein allocation predictions were to uncertainty in effective rate constants (*k*^eff^). To this end, we randomly perturbed effective rate constants by ±50% of their nominal values and maximized growth rate. Due to the considerable computational burden of many ME simulations, we limited the sensitivity analysis to glucose and acetate conditions. We ended up with 80 simulations that were still feasible after perturbations. We found that on average, the sensitivity to *k*^eff^ uncertainty was greatest at the proteome mass fraction level, decreased for metabolism, and was smallest for the growth rate. For protein mass fractions, the coefficient of variation (CV) of proteins across randomly perturbed simulations ranged between 0.073 to 3.9 (median of 0.31) and 0.086 to 8.9 (median of 0.62) for the Optimal and Generalist models, respectively (Fig. 2a). The Optimal model was significantly less sensitive to *k*^eff^ perturbations (Wilcoxon rank sum test, *P* < 2.2 × 10^−16^). Metabolic fluxes showed similar variability with CVs ranging from 0.29 to 4.8 (median of 0.38) and 0.28 to 9.7 (median of 0.61) for the Optimal and Generalist models, respectively (Fig. 2b). Again, the Optimal model was less sensitive to *k*^eff^ perturbations (Wilcoxon rank sum test, *P* = 1.0 × 10^−5^). Finally, growth rates varied with median CVs of 0.28 and 0.31 for Optimal and Generalist models, respectively (Fig. 2c), which was not significantly different between the two models (Wilcoxon rank sum test, *P* = 1). Therefore, we conclude that while uncertainty in *k*^eff^ can lead to wide variability in the expression of individual proteins, the effects are attenuated for metabolic fluxes and eventually growth rate.

## Discussion

One of the primary uses of ME models in previous studies has been to model optimal *E. coli* strains^27^. In particular, strains have been evolved in the laboratory while under environmental pressures designed to select for mutations that optimize for growth rate^28^. Such strains are highly useful in metabolic engineering applications where the goal is to produce a particular product efficiently^29^. However, the objective of all organisms is not necessarily to maximize growth rate. Many non-evolved laboratory strains or even pathogens have objectives that require allocation of proteomic and cellular resources to more than just growth rate^17^,^12^. To this end, we constrained a ME model using proteomics data collected under various conditions in order to better predict sub-optimal proteome allocation of “generalist” *E. coli*.

The ME model indicated that at growth rates as low as 0.1 h^−1^, up to 95% of the generalist proteome was not beneficial for growth. Much of the apparently wasted proteome was related to general stress response and “hedging” against environmental uncertainty. By integrating proteomics data into a ME model, we revealed the cellular cost of dedicating resources to maintaining this generalist proteome. We showed that proteomics data can be used to identify key proteome sector constraints and calibrate ME models. This approach can be extended in future work using sectors other than COGs, or determining novel sectors using the ME model itself. In particular, here we defined sector constraints capturing broad trends across many conditions, rather than fitting individual conditions. Yet, nearly all of the 15 minimal media examined showed improved predictions in terms of proteome allocation, growth rate, and metabolic fluxes. In future work, the sector constraints may be extended to include known regulation. For example, transcriptional regulation of carbon transport and utilization pathways has been well-studied for phosphotransferase system (PTS) sugars^30^ and non-PTS sugars^17^. Thus, it may be possible to combine regulatory models (e.g., by cyclic AMP-CRP) with ME models to model dynamic sector constraints in response to environmental changes such as carbon source availability or other environmental signals.

Further, we believe that the efforts here provide a framework moving forward for using novel data sets to tailor models to represent cellular objectives other than maximum growth rate. For example, with the various growth conditions in the data provided by Schmidt *et al*.^13^, we were able to place constraints on the proteome that allowed us to build a model for a generalist organism prepared for unfavorable conditions, as suggested by the significant enrichment of the constrained proteome sectors for the general stress response sigma factor *σ^S^*. Thus, new data sets that describe other experimental conditions or environmental pressures could be used to place additional constraints on the proteome using a ME model. Such experiments might measure the proteome across multiple nutrients under various stresses such as low or high pH^31^, iron limitation^32^, or exposure to reactive oxygen species^33^. This proteomics data could then be used to constrain the ME model’s stress response, thereby incorporating stress response into the objective function. Furthermore, with the increasing coverage and resolution of transcriptional regulatory interactions from Chromatin ImmunoPrecipitation (ChIP) experiments, it may ultimately be possible to integrate regulatory networks to proteome re-allocation. Combining data from proteomics, ChIP, metabolic flux analysis, physiological measurements (growth and exchange rates) and potentially metabolomics (at least metabolites involved in regulation) would provide the prerequisites for mechanistically modeling proteome allocation according to complex and multi-faceted cellular objectives.

One of the biggest challenges facing systems biology is to integrate new data types into genome-scale mathematical models to provide biologically meaningful phenotypic predictions. ME models provide mechanistic understanding of how metabolic flux states are linked to protein expression. Integrating data into a structured framework thus leads to an improved understanding of systems-level properties of organisms, suggesting a combined experimental and modeling approach to meet the “Big Data to Knowledge” challenge.

## Methods

### Simulating growth maximization using ME models

We used the latest published ME model of *E. coli* (iJL1678)^11^ for simulations consisting of nearly 80,000 biochemical reactions describing metabolism, transcription, and translation processes. To maximize growth rate in each growth condition, we used bisection (binary search) as in ref. 10 to maximize growth rate to six decimal points. Because ME Models are ill-conditioned^8^,^10^,^34^, we used the 128-bit (quad-precision) linear and nonlinear programming solver qMINOS 5.6^26^,^35^,^36^. (The soplex solver^37^ is another viable option, as it uses iterative refinement to achieve the needed numerical precision.) All qMINOS runs were performed with feasibility and optimality tolerances of 10^−20^. These tight tolerances were necessary because ME fluxes can vary by 15 orders of magnitude^26^.

### Computing generalist proteome allocation using sector constraints

The generalist ME model includes “sector constraints” in addition to the standard ME model formulation. The complete formulation of the optimization problem associated with the “generalist” ME model is the following:


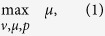



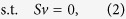



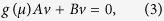



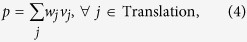



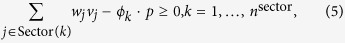



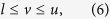


where *g*(*μ*) is a function of growth rate *μ, p* is the total simulated proteome mass, Translation is the set of translation fluxes, *n*^sector^ is the number of constrained sectors, Sector(*k*) is the index set of translation reactions in sector *k, w*_*j*_ is the molecular weight for protein (in g/mmol) *j, v*_*j*_ is the translation flux for protein *j* (in mmol/gDW/h), *ϕ*_*k*_ is the mass fraction of sector *k*, and *v, l* and *u* are the vectors of reaction fluxes, lower and upper flux bounds, respectively. “Optimal” ME models are formulated similarly, except without constraints (4) and (5).

Constraint (5) is the “sector constraint”, which constrains the summed mass fraction of a proteome sector to reach the specified amount. In this case, we constrained each sector by an inequality so that allocation to the constrained sector was greater than or equal to the measured mass fraction. The constraint is derived from the relation,


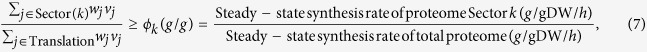


noting that the translation fluxes *v* are the rates of reactions that synthesize protein. The macromolecule Expression (E) matrix in the ME model enables the explicit computation of protein synthesis rate.

Our formulation can also be considered a more generalized extension of work by O’Brien *et al*.^12^, who determined a global parameter for the “un-utilized” and “under-utilized” protein expression using ME models. Here, we divided the proteome into functional sectors to a specified resolution or level of granularity and imposed constraints on several key sectors at this level.

Effective catalytic rate constants (*k*_eff_) were kept identical for both the Optimal and Generalist ME models, and were the same values as the original iJL1678 ME model^11^. Recall that effective rate constants *k*_eff_ and fluxes *v* are related as *v* = *k*_eff_*E*, where *E* is the enzyme concentration. Therefore, the maximum flux of a reaction is affected by proteome allocation across conditions.

### Comparing computed versus measured growth rates and proteomes

Computed growth rates *μ* were compared with those measured by Volkmer and Heinemann^38^. Computed proteome mass fractions were compared with those measured by Schmidt *et al*.^13^. Measured mass fractions were calculated using the measured protein masses (fg/cell). ME model mass fractions *f*_*i*_ were computed by weighting the translation flux (*v*_*i*_ in mmol/gram-dry-weight/h) of each protein by its molecular weight (*m*_*i*_): *f*_*i*_ = *m*_*i*_*v*_*i*_/∑_*j*∈*Translation*_*m*_*j*_*v*_*j*_, where *Translation* is the index set of translation reactions.

### Computing proteome size

ME models compute the protein fraction of dry cell weight, *P* (grams protein/gram dry weight). Because total protein synthesized was diluted by cell division, we have


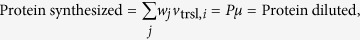


where *w*_*j*_ is the molecular weight of protein *j, v*_trsl,*j*_ is the translation flux of protein *j*, and *μ* is the growth rate (h^−1^). Therefore, *P* = ∑_*j*_*w*_*j*_*v*_trsl,*j*_/*μ*.

### Analyzing sensitivity to uncertainty in effective rate constants

We analyzed the sensitivity of proteome mass fraction, metabolic flux, and growth rate predictions to uncertainty in 1322 effective rate constants (*k*^eff^). We (uniformly) randomly perturbed these *k*^eff^ by ±50% of their original values in the published iJL1678 model^11^. Each randomly perturbed model was used to simulate growth maximization with and without sector constraints, as described in Methods.

### Enrichment analysis

Enrichment analysis was performed using hypergeometric test *p*-values, with *P* < 0.05 considered statistically significant.

### Proteome sector constraints

In this study, we used proteomics data from 15 minimal media conditions^13^ to define our sector constraints at the level of COGs^19^. The 15 conditions were the 4 chemostat conditions (dilution rates of 0.12, 0.20, 0.35, 0.50 h^−1^), and 11 minimal media (acetate, fructose, fumarate, galactose, glucosamine, glucose, glycerol, mannose, pyruvate, succinate, xylose). We chose the six sectors having at least 5% mass fraction across all conditions. The constrained mass fraction for each sector (*ϕ*_*i*_ in constraint (5)) was the minimum mass fraction across all conditions.

## Additional Information

**How to cite this article**: Yang, L. *et al*. Principles of proteome allocation are revealed using proteomic data and genome-scale models. *Sci. Rep.* **6**, 36734; doi: 10.1038/srep36734 (2016).

**Publisher’s note:** Springer Nature remains neutral with regard to jurisdictional claims in published maps and institutional affiliations.

## Supplementary Material

Supplementary Information

Supplementary Information

## Figures and Tables

**Table 1:** Sectors and their mass fractions defined from proteomics data and used to constrain the ME model.

Sector	Mass fraction
Amino acid transport and metabolism	0.115
Carbohydrate transport and metabolism	0.089
Cell wall/membrane/envelope biogenesis	0.068
Energy production and conversion	0.096
Posttranslational modification, protein turnover, chaperones	0.054
Translation, ribosomal structure and biogenesis	0.156

**Table 2:** Predicted protein percent of cell dry weight.

Condition	Optimal		Generalist	
	Growth rate, h^−1^	Protein, % dry weight	Growth rate, h^−1^	Protein, % dry weight
Acetate	0.672	60.1	0.344	70.0
Chemostat (µ = 0.12)	0.120	58.2	0.120	72.7
Chemostat (µ = 0.20)	0.200	58.0	0.200	71.0
Chemostat (µ = 0.35)	0.350	57.9	0.350	68.8
Chemostat (µ = 0.50)	0.500	58.1	0.500	66.7
Fructose	1.140	57.1	0.876	62.5
Fumarate	1.020	59.4	0.581	67.5
Galactose	1.020	57.8	0.760	62.8
Glucosamine	1.130	57.0	0.854	62.7
Glucose	1.140	57.4	0.886	62.4
Glycerol	1.140	57.2	0.821	63.4
Mannose	1.110	57.2	0.848	62.7
Pyruvate	1.010	58.7	0.950	61.9
Succinate	1.060	59.6	0.601	67.7
Xylose	1.020	57.9	0.734	63.6
