# Supplementary material for: Principles of proteome allocation are revealed using proteomic data and genome-scale models
Source: Sci Rep. 2016 Nov 18;6:36734. doi: 10.1038/srep36734 (PMC5114563; doi:10.1038/srep36734)
Supplement: Supplementary Information [file srep36734-s1.pdf]

Supplementary Material for “Principles of proteome allocation are revealed using proteomic data and genome-scale models”

***Laurence Yang*[1,5], *James T. Yurkovich*[1,2,5], *Colton J. Lloyd*[1], *Ali Ebrahim*[1], *Michael A. Saunders*[3], *Bernhard O. Palsson*[1,4]**
[1]*Department of Bioengineering, University of California, San Diego, La Jolla, California, USA.* [2]*Bioinformatics and Systems Biology Program, University of California, San Diego, La Jolla, California, USA.* [3]*Department of Management Science and Engineering, Stanford University, Stanford, California, USA.* [4]*Novo Nordisk Foundation Center for Biosustainability, The Technical University of Denmark, 2970 Hørsholm, Denmark.* [5]*These authors contributed equally to this work.*
email: palsson@ucsd.edu

Supplementary Tables

Table S1: Enrichment test of sectors for sigma factors. Ratio-sector denotes the number of overlapping genes relative to the sector, while ratio-σ is the number of overlapping genes relative to the genes controlled by the sigma factor.

Sector	σ factor	p-value	Ratio-sector	Ratio-σ
Amino acid transport and metabolism	D	3.475E-01	9.479E-01	6.798E-02
Amino acid transport and metabolism	S	6.656E-01	6.351E-01	6.617E-02
Amino acid transport and metabolism	H	9.051E-02	2.654E-01	7.921E-02
Amino acid transport and metabolism	N	2.457E-02	1.754E-01	9.227E-02
Amino acid transport and metabolism	E	9.893E-01	1.896E-02	2.817E-02
Amino acid transport and metabolism	F	5.993E-01	3.318E-02	6.481E-02
Carbohydrate transport and metabolism	D	4.067E-01	9.481E-01	4.351E-02
Carbohydrate transport and metabolism	S	1.965E-03	7.630E-01	5.086E-02
Carbohydrate transport and metabolism	H	4.794E-02	2.889E-01	5.516E-02
Carbohydrate transport and metabolism	N	8.776E-02	1.704E-01	5.736E-02
Carbohydrate transport and metabolism	E	9.872E-01	1.481E-02	1.408E-02
Carbohydrate transport and metabolism	F	5.012E-01	3.704E-02	4.630E-02
Cell wall/membrane/envelope biogenesis	D	3.540E-01	9.516E-01	4.011E-02
Cell wall/membrane/envelope biogenesis	S	3.268E-01	6.694E-01	4.099E-02
Cell wall/membrane/envelope biogenesis	H	4.039E-03	3.306E-01	5.799E-02
Cell wall/membrane/envelope biogenesis	N	7.358E-01	1.129E-01	3.491E-02
Cell wall/membrane/envelope biogenesis	E	1.975E-01	6.452E-02	5.634E-02
Cell wall/membrane/envelope biogenesis	F	4.262E-01	4.032E-02	4.630E-02
Energy production and conversion	D	9.692E-02	9.688E-01	4.215E-02
Energy production and conversion	S	4.674E-02	7.188E-01	4.543E-02
Energy production and conversion	H	9.480E-01	1.719E-01	3.112E-02
Energy production and conversion	N	2.763E-01	1.484E-01	4.738E-02
Energy production and conversion	E	1.000E+00	0.000E+00	0.000E+00
Energy production and conversion	F	4.538E-01	3.906E-02	4.630E-02
Posttranslational modification, protein turnover, chaperones	D	9.594E-01	8.977E-01	2.685E-02
Posttranslational modification, protein turnover, chaperones	S	5.366E-01	6.477E-01	2.815E-02
Posttranslational modification, protein turnover, chaperones	H	1.759E-04	3.977E-01	4.950E-02
Posttranslational modification, protein turnover, chaperones	N	8.123E-01	1.023E-01	2.244E-02
Posttranslational modification, protein turnover, chaperones	E	2.889E-05	1.591E-01	9.859E-02
Posttranslational modification, protein turnover, chaperones	F	8.150E-01	2.273E-02	1.852E-02
Translation, ribosomal structure and biogenesis	D	7.633E-04	9.932E-01	4.997E-02
Translation, ribosomal structure and biogenesis	S	3.946E-02	7.162E-01	5.235E-02
Translation, ribosomal structure and biogenesis	H	9.491E-07	3.986E-01	8.345E-02
Translation, ribosomal structure and biogenesis	N	9.979E-01	6.081E-02	2.244E-02
Translation, ribosomal structure and biogenesis	E	9.689E-01	2.027E-02	2.113E-02
Translation, ribosomal structure and biogenesis	F	1.000E+00	0.000E+00	0.000E+00

Table S2: Computed and measured growth rates by the optimal and generalist ME models. $\Delta\mu$ and $\%\Delta\mu$ denote absolute and percent change in growth rate, respectively. Rank denotes the rank of $\Delta\mu$, ordered from largest to smallest difference.

Condition	Optimal	Generalist	Measured	$\Delta\mu$	% $\Delta\mu$	Rank
Acetate	0.672	0.344	0.300	-0.328	-48.857	1
Fructose	1.138	0.876	0.650	-0.262	-23.000	9
Fumarate	1.020	0.581	0.420	-0.439	-43.014	3
Galactose	1.025	0.760	0.260	-0.265	-25.821	6
Glucosamine	1.128	0.854	0.460	-0.275	-24.337	7
Glucose	1.136	0.886	0.580	-0.249	-21.947	10
Glycerol	1.141	0.821	0.470	-0.320	-28.011	4
Mannose	1.115	0.848	0.470	-0.267	-23.951	8
Pyruvate	1.008	0.950	0.400	-0.058	-5.773	11
Succinate	1.056	0.601	0.440	-0.454	-43.042	2
Xylose	1.018	0.734	0.550	-0.284	-27.912	5

Table S3: Mass fraction difference between computed efficient and measured proteomes.

Growth rate (h^{-1})	Subsystem	Mass fraction (exp)	Mass fraction (optimal)	Percent difference (%)
0.120	Carbohydrate Metabolism	0.299	0.015	-95
0.200	Carbohydrate Metabolism	0.276	0.024	-91
0.350	Carbohydrate Metabolism	0.254	0.043	-83
0.500	Carbohydrate Metabolism	0.208	0.061	-71

Table S4: Significance of shifts in computed metabolic fluxes between optimal and generalist ME models, where p.wilcox is the p-value for the two-sided Wilcoxon rank-sum test, while p.greater and p.less are p-values for the Wilcoxon rank-sum test with alternative hypothesis that the optimal model has larger or smaller metabolic fluxes than the generalist model, respectively, and n is the number of fluxes in each subsystem excluding reactions having zero flux in both models.

cond	Supersystem	p.wilcox	p.greater	p.less	n
Glucose	Membrane Transport	4.802E-01	2.401E-01	7.603E-01	145
Glucose	Lipid Metabolism	3.085E-09	1.542E-09	1.000E+00	86
Glucose	Cell Wall/Membrane/Envelope Metabolism	5.663E-02	2.831E-02	9.719E-01	90
Glucose	Nucleotide, Cofactor and Prosthetic Group Metabolism	5.438E-03	2.719E-03	9.973E-01	273
Glucose	AA and Carbohydrate Metabolism	3.221E-02	1.610E-02	9.839E-01	536
Glucose	Energy Production and Conversion	2.055E-01	1.027E-01	8.990E-01	40
Glucose	Other	5.615E-02	2.807E-02	9.721E-01	100
Glucose	Inorganic Ion Transport and Metabolism	1.826E-01	9.132E-02	9.090E-01	114
Acetate	Membrane Transport	9.063E-02	4.532E-02	9.548E-01	129
Acetate	Lipid Metabolism	2.197E-13	1.099E-13	1.000E+00	86
Acetate	Cell Wall/Membrane/Envelope Metabolism	7.333E-03	3.666E-03	9.964E-01	92
Acetate	Nucleotide, Cofactor and Prosthetic Group Metabolism	1.454E-04	7.270E-05	9.999E-01	264
Acetate	AA and Carbohydrate Metabolism	3.124E-05	1.562E-05	1.000E+00	515
Acetate	Energy Production and Conversion	3.322E-02	1.661E-02	9.839E-01	34
Acetate	Other	1.648E-03	8.239E-04	9.992E-01	104
Acetate	Inorganic Ion Transport and Metabolism	1.755E-02	8.773E-03	9.913E-01	108

Supplementary Figures

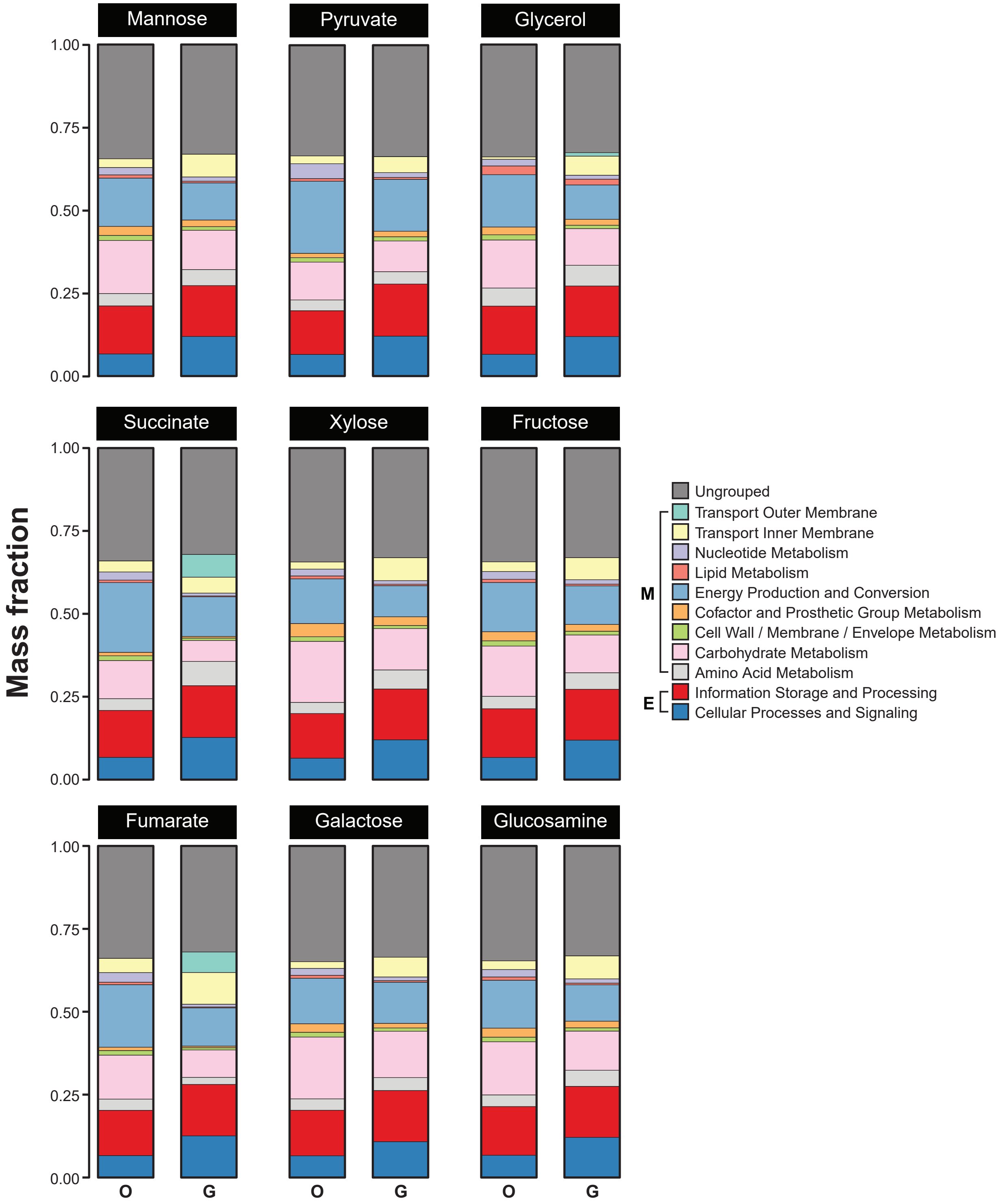

Figure S1: Global proteome allocation computed by optimal (O) and generalist (G) ME models for Metabolic (M) and Expression (E) systems in minimal media with different carbon sources.

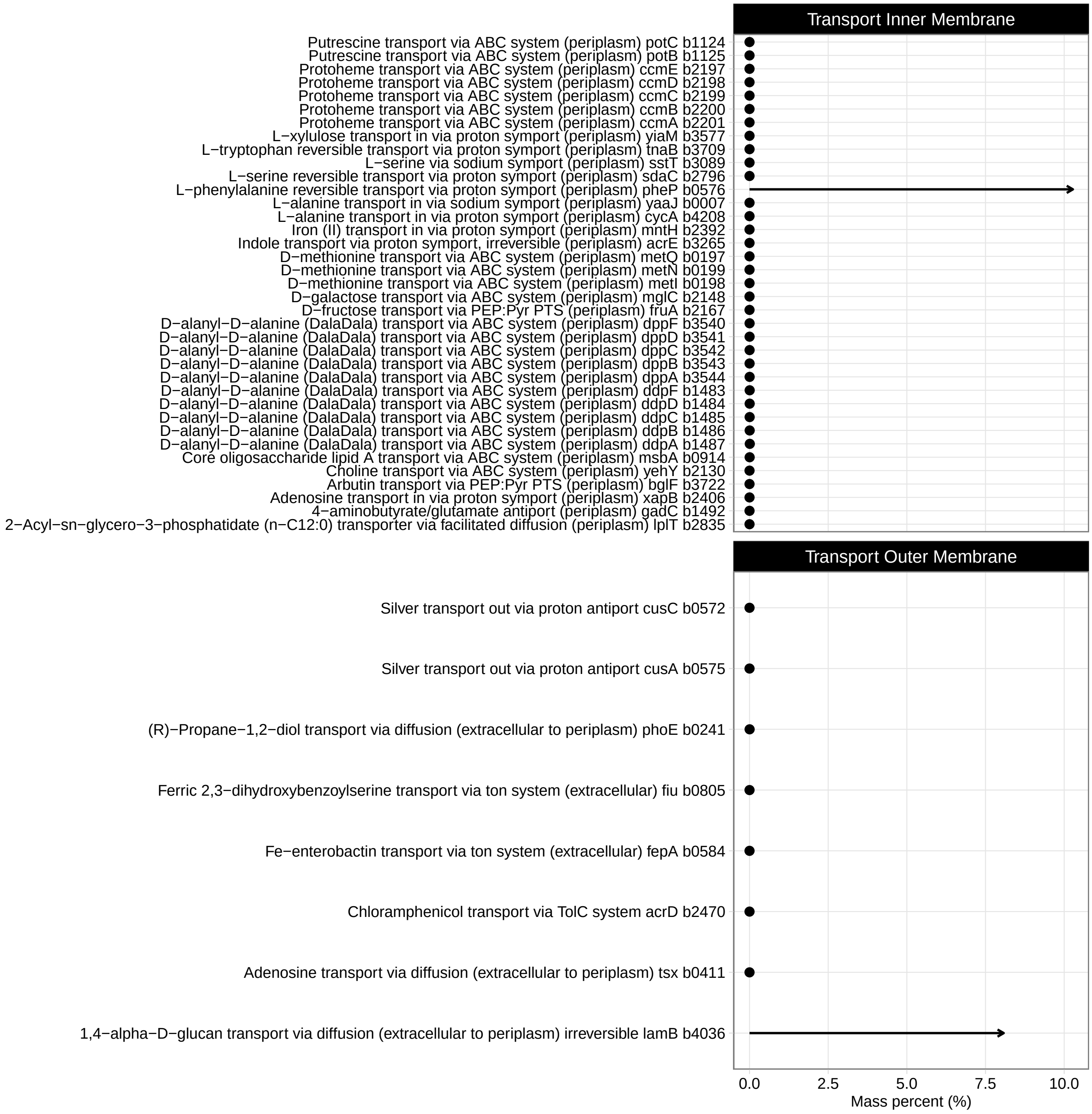

Figure S2: Shift in proteome allocation of membrane transport proteins. Arrows point from the mass fraction of each protein in the optimal ME model to that in the generalist ME model. Proteins with mass percent changes below 0.05% are shown as points.

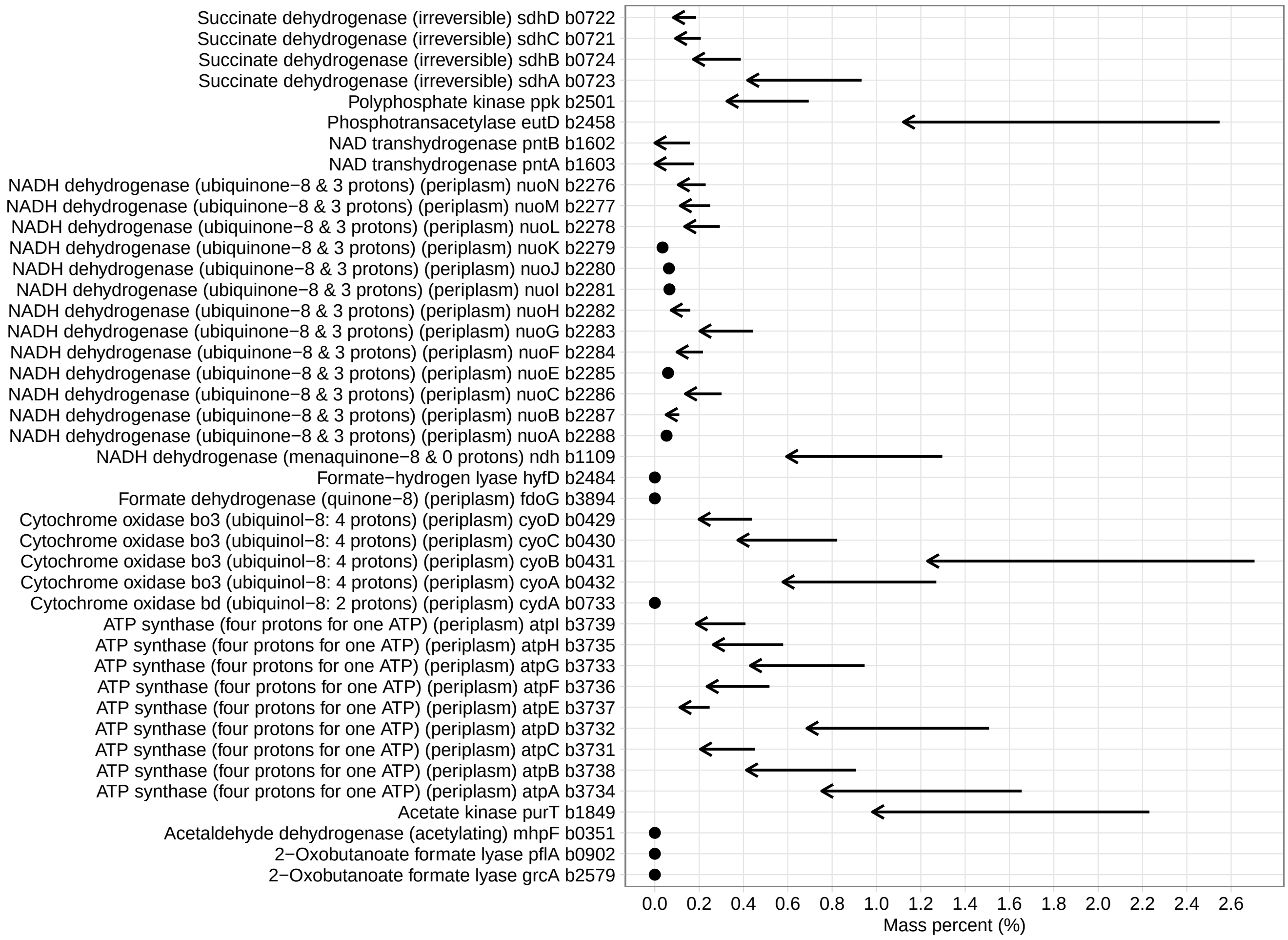

Figure S3: Shift in proteome allocation of proteins in the "energy production and conversion" sector. Arrows point from the mass fraction of each protein in the optimal model to that in the generalist ME model. Proteins with mass percent change below 0.05% are shown as points.

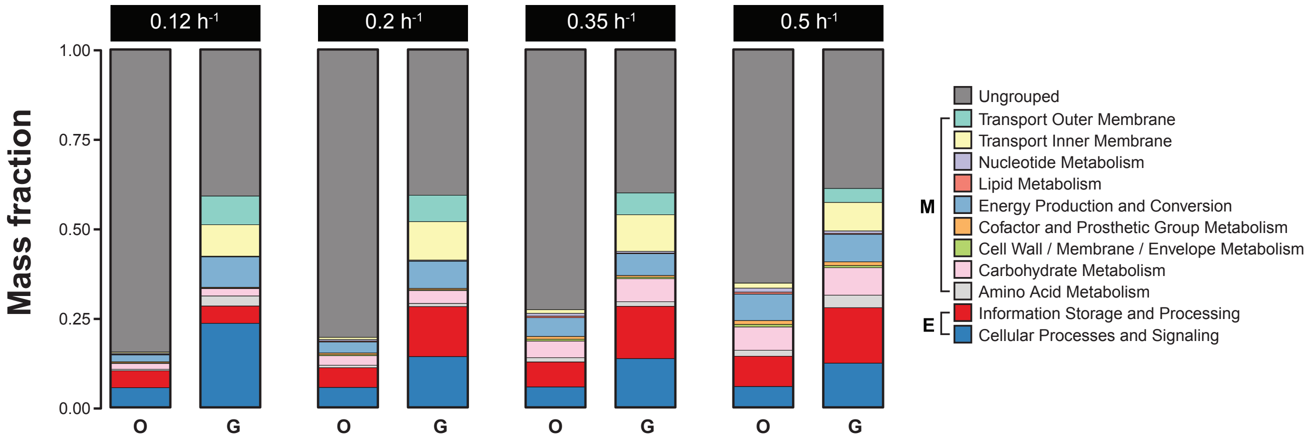

Figure S4: Global proteome allocation computed by optimal (O) and generalist (G) ME models for Metabolic (M) and Expression (E) systems in chemostat conditions with dilution rates ranging from 0.12 h^{-1} to 0.5 h^{-1}.

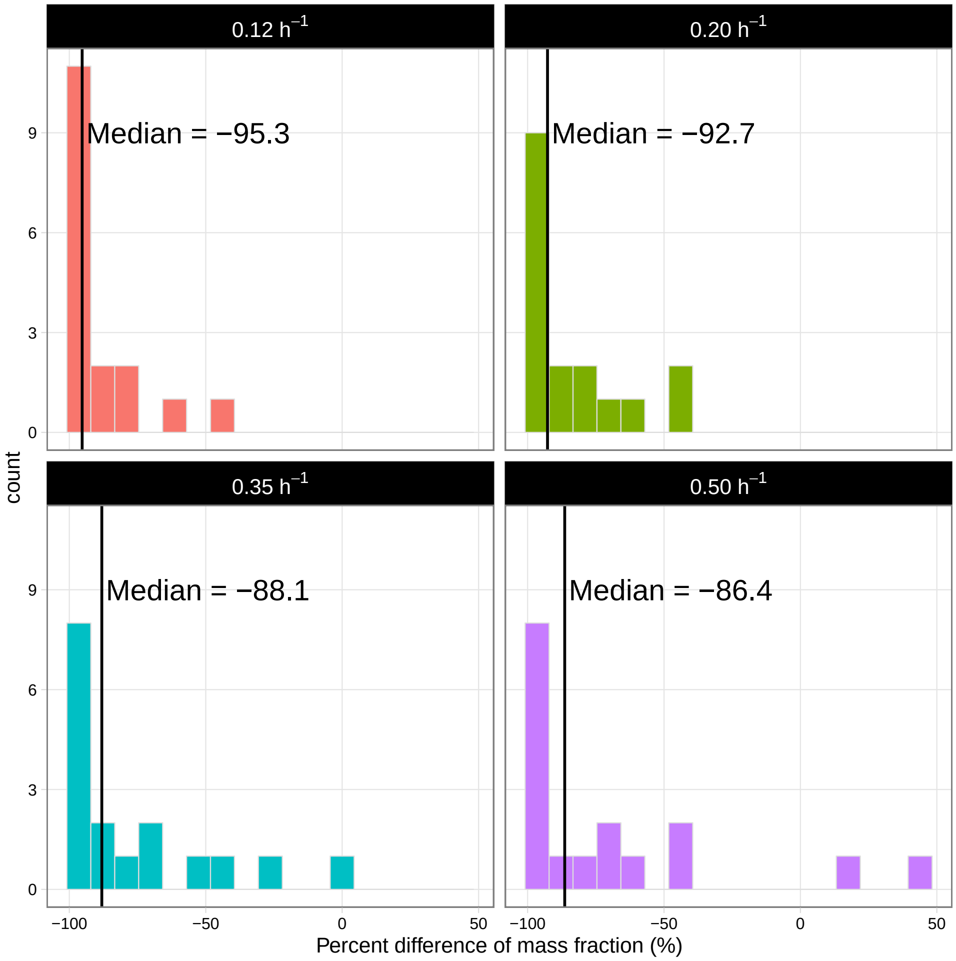

Figure S5: Histograms of protein mass fraction differences between measured proteins and those computed using the optimal ME model in chemostat conditions with dilution rates ranging from 0.12 h^{-1} to 0.5 h^{-1}. Mass fractions were compared at the level of metabolic and expression systems, as described in Fig. 1 of the main article.

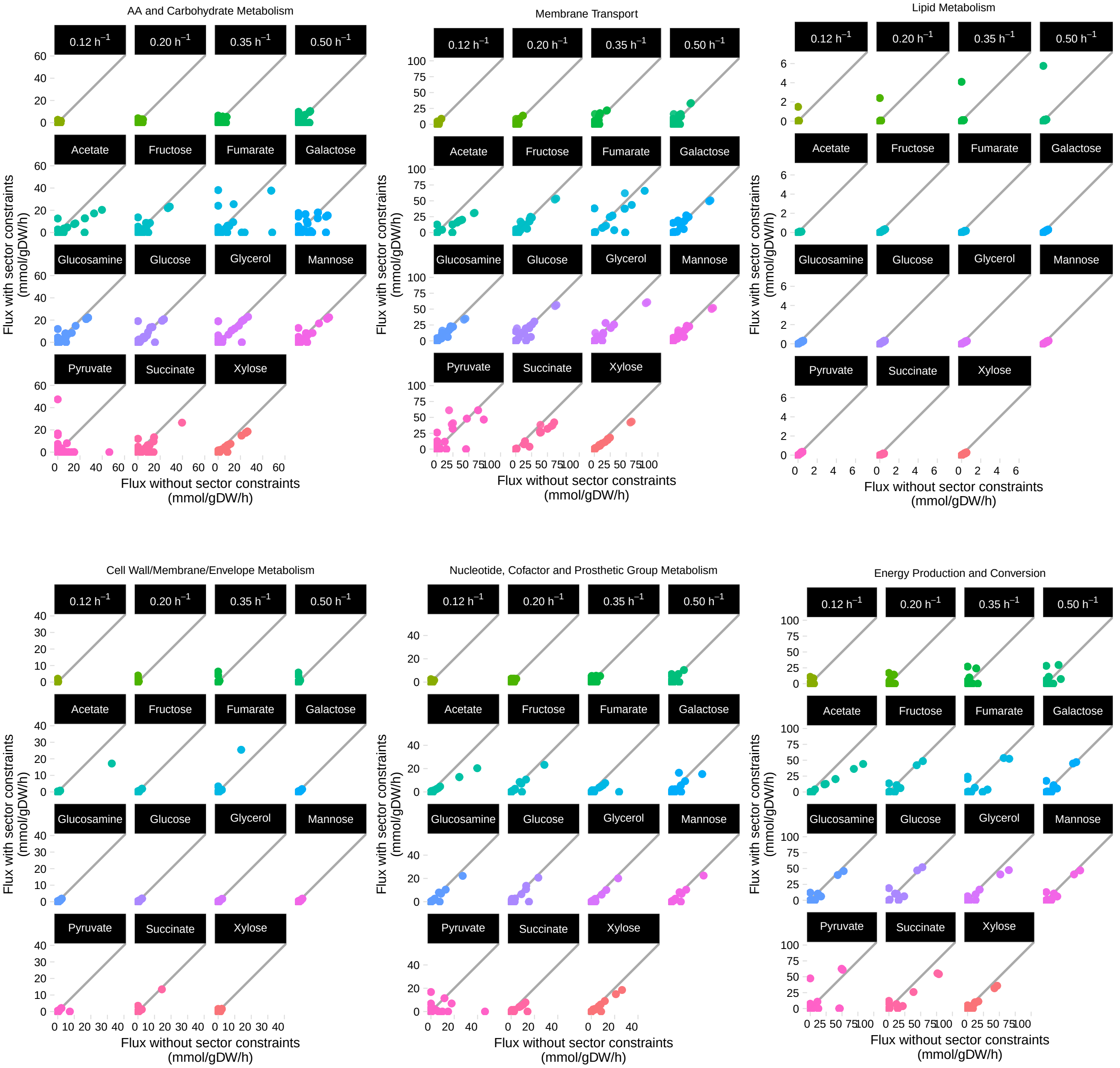

Figure S6: Metabolic flux shifts between optimal and generalist ME models in 15 minimal media conditions. Chemostat conditions are labeled with their corresponding dilution rates ranging from 0.12 h^{-1} to 0.5 h^{-1}.

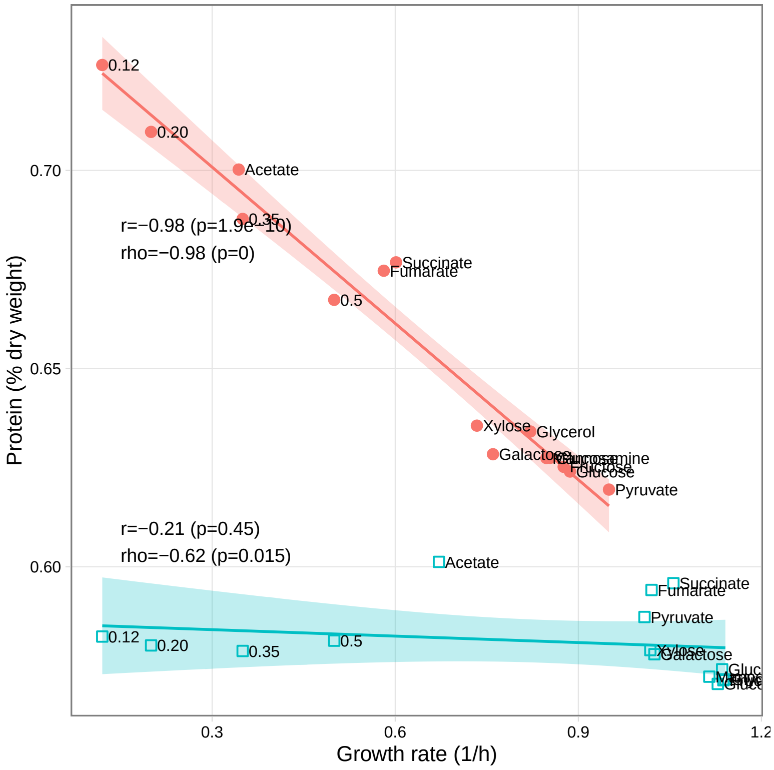

Figure S7: Computed protein size (% cell dry weight) versus computed growth rate. Blue squares: Optimal model. Red circles: Generalist model.

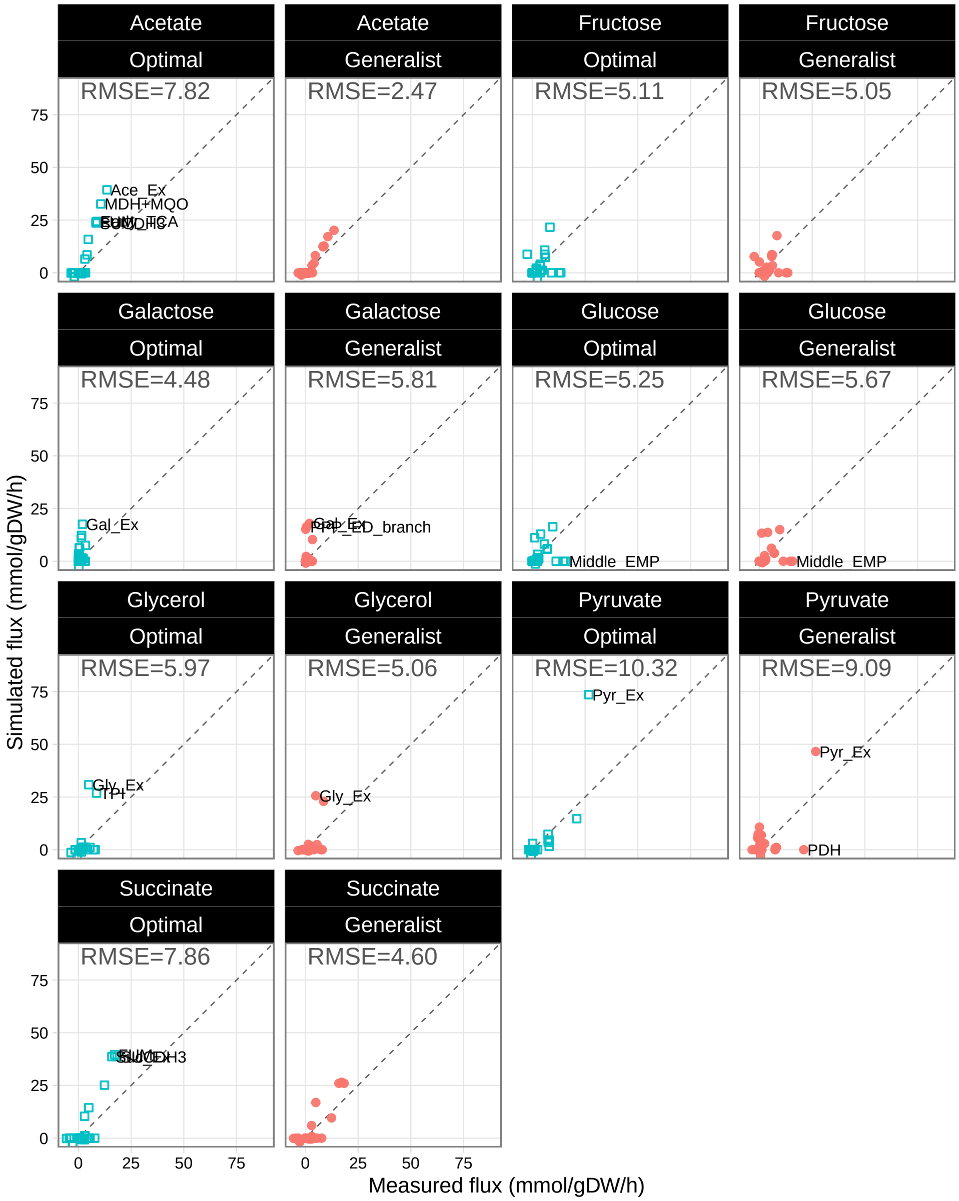

Figure S8: Comparison of computed fluxes with those determined using metabolic flux analysis by Gerosa et al.[1]

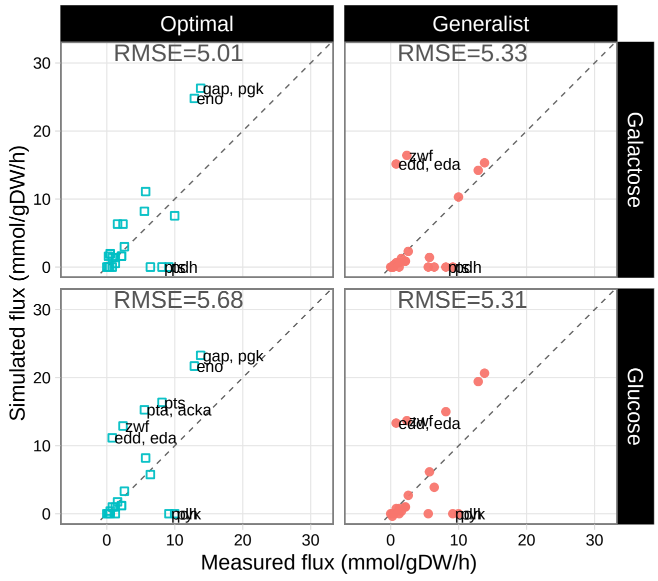

Figure S9: Comparison of computed fluxes with those determined using metabolic flux analysis by van Rijsewijk et al.[2]

References

1. Luca Gerosa, Bart RB Haverkorn van Rijsewijk, Dimitris Christodoulou, Karl Kochanowski, Thomas SB Schmidt, Elad Noor, and Uwe Sauer. Pseudo-transition analysis identifies the key regulators of dynamic metabolic adaptations from steady-state data. *Cell Systems*, 1(4):270–282, 2015.

2. Bart RB Haverkorn van Rijsewijk, Annik Nanchen, Sophie Nallet, Roelco J Kleijn, and Uwe Sauer. Large-scale 13c-flux analysis reveals distinct transcriptional control of respiratory and fermentative metabolism in escherichia coli. *Mol Syst Biol*, 7(1):477, 2011.
